# Dr. John L. Eddy

**Published:** 1902-05

**Authors:** 


### OBITUARY NOTES.

---

Dr. John L. Eddy, of Olean, N. Y., died at his home in that city of apoplexy, April 5, 1892, aged 73. He was a graduate of Castleton, Vt., Medical College, in 1854, and was one of the most prominent physicians in his region. He was a surgeon of considerable eminence and was attached to the medical staff of the Erie Railway. He had been also a pension examiner for many years. His son, a young physician of promise, died a few years ago.

